# Genome wide CNV analysis reveals additional variants associated with milk production traits in Holsteins

**DOI:** 10.1186/1471-2164-15-683

**Published:** 2014-08-15

**Authors:** Lingyang Xu, John B Cole, Derek M Bickhart, Yali Hou, Jiuzhou Song, Paul M VanRaden, Tad S Sonstegard, Curtis P Van Tassell, George E Liu

**Affiliations:** Animal Genomics and Improvement Laboratory, BARC, USDA-ARS, Beltsville, Maryland 20705 USA; Department of Animal and Avian Sciences, University of Maryland, College Park, Maryland 20742 USA; Beijing Institute of Genomics, Chinese Academy of Sciences, Beijing, 100029 China

**Keywords:** Copy number variation (CNV), dPTA, Association, Milk production traits

## Abstract

**Background:**

Milk production is an economically important sector of global agriculture. Much attention has been paid to the identification of quantitative trait loci (QTL) associated with milk, fat, and protein yield and the genetic and molecular mechanisms underlying them. Copy number variation (CNV) is an emerging class of variants which may be associated with complex traits.

**Results:**

In this study, we performed a genome-wide association between CNVs and milk production traits in 26,362 Holstein bulls and cows. A total of 99 candidate CNVs were identified using Illumina BovineSNP50 array data, and association tests for each production trait were performed using a linear regression analysis with PCA correlation. A total of 34 CNVs on 22 chromosomes were significantly associated with at least one milk production trait after false discovery rate (FDR) correction. Some of those CNVs were located within or near known QTL for milk production traits. We further investigated the relationship between associated CNVs with neighboring SNPs. For all 82 combinations of traits and CNVs (less than 400 kb in length), we found 17 cases where CNVs directly overlapped with tag SNPs and 40 cases where CNVs were adjacent to tag SNPs. In 5 cases, CNVs located were in strong linkage disequilibrium with tag SNPs, either within or adjacent to the same haplotype block. There were an additional 20 cases where CNVs did not have a significant association with SNPs, suggesting that the effects of those CNVs were probably not captured by tag SNPs.

**Conclusion:**

We conclude that combining CNV with SNP analyses reveals more genetic variations underlying milk production traits than those revealed by SNPs alone.

**Electronic supplementary material:**

The online version of this article (doi:10.1186/1471-2164-15-683) contains supplementary material, which is available to authorized users.

## Background

Milk production is an economically important sector of global agriculture and much attention has been paid to improve milk performance-related traits in cattle. Various methods have been employed to identify significant genetic markers for milk production. These methods include quantitative trait loci (QTL) mapping with different mapping designs and genome-wide association studies with a variety of statistical tests. Many QTL related to milk production traits have been reported using different populations and DNA markers, such as microsatellite [1–4] and SNPs [5–11]. The identification of QTL and investigation of genetic and molecular mechanisms underlying those QTL may result in more efficient animal selection and increased rates of genetic progress. However, most of these cattle QTL studies did not identify the casual variant, which is useful information for breeding applications to avoid losses in accuracy because of recombination between associated QTL markers and the actual quantitative trait nucleotide (QTN). Only a few casual mutations within genes of known function, such as *DGAT* [3, 12, 13], *ABCG* [4] and *GRH* [14, 15], have been identified with their large effects on milk production validated. Additionally, most of those GWAS studies concluded that SNP may only explain a small portion of genetic variance. Alternative frameworks to explain the missing heritability of complex traits were proposed [16].

Genomic structural variants are comprised mainly of copy number variations (CNVs) in the form of large-scale insertions and deletions, as well as inversions and translocations [17]. Compared to SNPs, CNVs involve more genomic sequence and have potentially greater effects, including changing gene structure and dosage, alternating gene regulation and exposing recessive alleles [18]. Human and mouse studies have found that CNVs capture 18 to 30% of the genetic variation in gene expression [19, 20]. Those CNVs were shown to be important in both normal phenotypic variability and disease susceptibility. In livestock, most CNV studies have used limited CNV detection methods, including CGH arrays, SNP arrays, and next generation sequencing [21–31]. Our previous studies have indicated that some CNVs could be associated with resistance or susceptibility to gastrointestinal nematodes in Angus cattle [32] and residual feed intake in Holstein cows [33]. Moreover, Glick et al. identified a CNV associated with fertility in Israeli Holsteins [34]. A recent study reported a 660 kb deletion with antagonistic effects on fertility and milk production in Nordic Red cattle [35]. Kadri et al. previously reported linkage disequilibrium (LD) between one deletion and its neighboring SNPs in Holsteins cattle [36]. However, no study has reported about genome wide CNV association directly with milk production traits. Furthermore, no systematic study of the relationship between CNVs and SNPs in the bovine genome has been published.

In this study, we reported a systematic CNV association analysis with milk production traits in 26,362 US Holsteins. Thirty-four CNVs have been identified as significantly associated with milk production traits using an association test, and most of them overlap known QTL. Haplotype analysis for associated CNVs and neighboring SNPs produced further evidence that CNVs provide additional information that is not captured by SNPs alone. Therefore, CNVs could be utilized as additional molecular markers for use in genetic improvement programs.

## Methods

### Samples

Holstein bulls and cows (26,362 samples) were genotyped using the Illumina BovineSNP50 array version 1 (Illumina Inc., San Diego, CA). Genotypes of those animals have been included in the routine genomic evaluation program for the United States and Canada since 2009 [37, 38]. The main source of extracted DNA for bulls was semen from the Cooperative Dairy DNA Repository and from the National Center for Genetic Resources Preservation, ARS, USDA (Fort Collins, CO). The research did not involve any experiment on animals and for this reason no ethics approval was necessary.

### Phenotypic and dPTA values

Traditional predicted transmitting abilities (PTAs) for five production traits, including milk yield (MY), fat yield (FY), protein yield (PY), fat percentage (FP), and protein percentage (PP), were calculated by USDA ARS AIPL (Beltsville, MD). Those PTA are predicted additive genetic effects after removing fixed non-genetic effects, and the reliabilities of the PTA were used to quantify the amount of information available for different individuals [39, 40]. De-regressed PTAs (dPTA) were computed as in Garrick et al. [40] by dividing PTA by their squared reliability [dPTA = PTA/(reliability)^2^]. The dPTA were used as the phenotypes for genome-wide association studies.

### CNV segmentation and genotyping

The intensity data of 56,947 SNP probes were generated by Illumina BovineSNP50 arrays. We imported Log R Ratios (LRR) from the GenomeStudio software into Golden Helix SNP & Variation Suite (SVS) 7.7 (Golden Helix Inc., Bozeman, MT, USA) using its DSF Export Plug-In 4.1.

A total of 48,669 SNPs were mapped onto the *Bos taurus* genome assembly UMD 3.1 (https://ccb.jhu.edu/bos_taurus_assembly.shtml) within 29 autosomes.

To normalize the LRR, we used the default GC correlation file (GC Reference bos_taurus_UMD3.1.gc_digest.dsf) to correct for the waviness contributed by GC content. We then utilized the copy number analysis module (CNAM) under the multivariate option to segment chromosomes with a maximum of 20 segments per window, a minimum of 3 markers per segment, and a significance level of p = 0.01 for pairwise permutations (n = 1,000) as described previously [41].

### PCA-corrected association testing

In Golden Helix SVS 7.7, a linear regression under the additive genetic model was employed to identify CNVs associated with each production trait with the option of Full Scan Permutations (10,000 permutations). We used the principal component analysis (PCA) option to correct batch effects/stratification of the test input data. Significant CNVs were counted at the significance level of (p-value < 0.05) after FDR correction.

### Association tests based on SNPs

Association tests were carried out using the linear model plugin of SVS 7.7 software with PCA correction. Significant SNPs were detected when their adjusted p-values passed the threshold of p < 1 × 10^-8^ after FDR correction.

### Haplotype block analysis

To investigate linkage disequilibrium (LD) patterns in the regions containing associated CNVs, pairwise LD statistics D’ and r^2^ were calculated using Haploview (Version 4.2) [42] and the LD blocks were defined by the criteria of Gabriel et al. [43]. Qanbari et al. [44] presented a linkage disequilibrium (LD) map for Holsteins based on the same Bovine SNP50 array. Based on their maximum haplotype block length (1.26 Mb), the regions considered for LD analysis were extended 25 SNPs on the both upstream and downstream directions of each CNV (i.e., 1.26 Mb/average marker spacing of 50 kb).

### Relationship between significantly associated CNVs and significantly associated SNPs

We classified all 82 significant combinations of traits and CNVs (less than 400 kb in length and p values < 0.05 after FDR correction as shown in bold and italic in Table 1) in the following way. First, when a significant SNP directly overlapped with a CNV by genomic coordinate, we called the situation as “IN”. If a SNP was directly adjacent to a CNV, we called it “IN*”. When a SNP was the second SNP next to a CNV, we called it “IN**”. Any SNPs beyond the neighboring 2 positions of a CNV were not considered in this comparison. We then evaluated the linkage relationship between CNVs and neighboring SNPs. When a CNV was in the same haploblock with at least one significant SNP, we called it “LD”. When a CNV was adjacent to a haploblock which contains at least one significant SNP, we called it “LD*”. In contrast, when a CNV was in a haploblock which contained no significant SNP, we called it “LD NS”. When a CNV was adjacent to a haploblock which contained no significant SNP, we called it “LD* NS”. For complicated situation where both “IN” and “LD” existed, “IN” was considered first and used instead of “LD” as the category. Finally, when a CNV did not overlap with any SNPs and was not in a haploblock, we called it “NN”.

**Table 1:** **Relationship classification between trait associated CNVs and their neighboring SNPs for 82 combinations of trait and CNVs**

CNV#	P value after FDR correction					Tagged by SNPs					Breakpoint	PennCNV
	MY	FY	PY	FP	PP	MY	FY	PY	FP	PP		Support
1	***2.56E-06***	***2.12E-08***	***0.0246***	0.0657	***3.96E-21***	IN*	IN*	IN*		NN		
2	***8.53E-07***	***1.32E-07***	***0.0020***	0.8913	***1.46E-09***	IN	IN	IN		NN		Yes
3	0.0810	0.1375	0.9468	0.6499	***1.32E-32***					IN*		
4	***1.26E-09***	***1.99E-08***	***4.06E-09***	0.1334	0.0756	IN**	IN**	IN**				Yes
5	0.2506	***0.0473***	0.2410	0.0758	***0.0008***		IN*			IN*	Yes	Yes
6	***0.0008***	***1.91E-06***	***4.95E-05***	***0.0004***	***6.95E-22***	NN	NN	IN	NN	NN		Yes
7	***0.0020***	***0.0001***	***0.0418***	0.2851	***1.38E-05***	IN**	IN**	NN		IN**		Yes
8	***9.03E-10***	***5.38E-10***	***1.61E-06***	1.0000	***9.35E-05***	IN*	IN*	IN*		IN*		Yes
9	***0.0005***	***2.50E-06***	0.2701	***0.0049***	***9.47E-18***	IN**	IN**		NN	IN		
10	***9.29E-12***	***8.38E-12***	***2.23E-07***	1.0000	0.0997	IN	IN	IN*				
11	***3.81E-05***	***6.52E-05***	***0.0345***	0.7610	***0.0037***	IN*	IN*	IN*		IN	Yes	Yes
12	***0.0006***	0.1336	0.5275	***3.70E-09***	***1.52E-22***	IN*			IN*	NN		Yes
14	0.1484	0.5341	0.7490	0.0700	***0.0371***					IN*	Yes	
16	0.2552	0.8739	***0.0352***	***0.0033***	0.2416			NN	LD*			Yes
17	***6.96E-07***	***2.87E-06***	***2.99E-05***	0.4144	0.9401	IN*	IN**	IN*				Yes
18	0.0890	***2.02E-05***	***0.0062***	***1.79E-13***	***6.21E-15***		LD	LD	IN* + LD	IN*	Yes	Yes
19	***0.0032***	***3.53E-05***	0.2698	***0.0061***	***0.0035***	IN*	IN*		IN*	IN	Yes	
20	0.9468	0.8614	0.6384	1.0000	***0.0286***					NN		
21	0.0798	0.1692	0.5656	0.3360	***0.0433***					IN + LD		Yes
22	0.6780	0.4652	0.3753	0.9048	***0.0005***					NN	Yes	Yes
23	***0.0065***	***0.0209***	***0.0008***	0.5674	***7.60E-05***	NN	IN**	IN**		NN		
24	0.0616	0.0844	0.6569	0.8919	***6.98E-09***					NN	Yes	Yes
25	***4.43E-08***	***1.58E-08***	***1.94E-05***	0.9206	0.4082	IN	IN**	IN**				
26	0.8385	0.7831	0.8926	0.9465	***2.51E-16***					IN + LD		Yes
28	***0.0016***	***1.70E-08***	***0.0008***	***2.21E-13***	***0.0002***	LD* NS	LD* NS	LD* NS	IN* + LD	LD*	Yes	Yes
29	***8.09E-07***	***2.03E-07***	***0.0007***	0.7631	***3.02E-05***	IN + LD	IN + LD	IN + LD		NN		Yes
31	0.1197	***0.0159***	0.1152	0.0773	***3.15E-05***		IN			IN**	Yes	Yes
32	***9.08E-09***	***3.95E-08***	***0.0001***	0.5293	***4.20E-07***	LD NS	NN	LD		IN + LD		

CNV27, CNV33 to CNV37 were not considered due to their large sizes (> 400 kb). For CNVs less than 400 kb in length, their p values < 0.05 after FDR correction were shown in bold and italic. For the meanings of IN*, IN**, LD*, please refer to the main text.

## Results and discussion

### Trait properties and correlations

We selected five traits related to milk production for an association analysis: milk yield (MY), fat yield (FY), protein yield (PY), fat percentage (FP), protein percentage (PP). The descriptive statistics of PTA, including reliability and heritability, are given in Additional file 1: Figure S1 and Additional file 2: Figure S2, respectively. Pearson correlation coefficients for all pairs of traits are provided in Table 2. As expected, the three yield traits (MY, FY and PY) are strongly and positively correlated. The two percentage traits (FP and PP) are positively correlated with each other, but are negatively correlated with the yield traits.

**Table 2:** **Pairwise Pearson correlation coefficients for all pairs of traits**

	Milk Yield	Fat Yield	Protein Yield	Fat Percentage	Protein Percentage
Milk Yield	-	0.7181	0.9040	-0.3285	-0.3596
Fat Yield	0.7181	-	0.7938	0.4206	0.0581
Protein Yield	0.9040	0.7938	-	-0.1012	0.0723
Fat Percentage	-0.3285	0.4206	-0.1012	-	0.5470
Protein Percentage	-0.3596	0.0581	0.0723	0.5470	-

### CNV segmentation and genotyping

In contrast to conventional CNV discovery studies, which try to detect as many variable regions as possible, this CNV-based GWAS is intended to identify the common CNVs shared among samples in order to detect associations with common diseases or traits. Using the multivariate method of CNAM in SVS, a total of 2,626,669 distinct segments were detected in the 26,362 samples. After merging across samples, 99 nonredundant CNVs were left for subsequent association test (Additional file 3: Table S1). Within these 99 segments, each sample was genotyped (i.e., called as loss, neutral or gain event) according to a three-state model with strict threshold levels of marker mean ± 0.5. Since the multivariate CNAM method was developed to identify common CNVs, only those segments with frequencies above 0.4% were retained for further analysis in order to filter away false positive calls. A total of 39 CNVs ranging in size from 45,109 bp to 7.16 Mb were retained (frequency > 0.04). These 39 CNVs have an estimated average size and SNP count of 962.71 kb and 18.4, respectively.

### CNV association analyses

**Figure 1:**
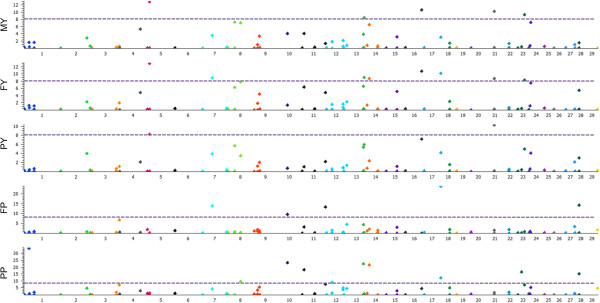
**Manhattan plots of associated CNVs for five milk production traits (Milk Yield, Fat Yield, Protein Yield, Fat Percentage and Protein Percentage) using linear regression model.** Negative log10-transformed P values from a genome-wide scan are plotted against genomic coordinates on 29 autosomal chromosomes.

A total of 26,362 Holstein cattle were employed to test associations between CNVs and dPTA data. Using a linear regression, we identified a total of 34 CNVs that were significantly associated with at less one trait (Figure 1 and Additional file 4: Table S2). Among those 34 associated CNVs, the CNV with the highest frequency (96.03%) was found at chr14:11,250,157-11,307,423, while the CNV with the lowest frequency (2.09%) was localized at chr29:46,099,425-51,502,868 (Additional file 4: Table S2). After we removed 6 CNVs larger than 400 kb (CNV27, CNV33 to CNV37), we compared the 28 remaining CNVs with 5 previously published results [23, 26, 27, 45, 46]. We found 23 out of these 28 CNVs (82.1%) were also previously reported, and the 5 non-overlapping CNVs (CNV4, 5, 8, 9 and 23) had high frequencies (ranging from 30 to 70%) and large marker mean changes (ranging from -0.56 to -1.31, deviated from 0), suggesting they are probably real CNVs. Three CNVs: CNV6 (chr13:70,496,054-70,623,303), CNV28 (chr7:42,700,425-42,788,788), and CNV33 (chr17:73,055,503-75,058,715) had P values < 0.05 after FDR correction for all five traits. Their frequencies were 66.88%, 16.95% and 7.55%, respectively (Additional file 4: Table S2). Another 12 CNVs were significantly associated with four traits. The overlapping relationship of these associated CNVs among 5 traits was shown in a Venn diagram (Additional file 5: Figure S3).

We then overlapped these 34 associated CNVs with the reported cattle QTL as reported by the Animal QTL database at http://www.animalgenome.org/QTLdb/cattle.html [47]. When considered together, 21 of the 34 significant CNVs overlapped with at least one of the known QTL for milk production. Among these 34 CNVs, we found 15, 15, 14, 7 and 6 CNVs overlapping with QTL for MY, FY, PY, FP and PP traits, respectively (Additional file 4: Table S2). Based on the UMD 3.1 assembly, Additional file 4: Table S2 summarizes these 34 CNV regions, bovine RefSeq gene annotations within the CNV regions (1X), and flanking regions (3X: extended regions by one CNV length in both downstream and upstream directions).

### CNVs significantly associated with milk traits

Of the 34 CNVs, 19, 23 and 18 were significantly associated with MY, FY and PY, respectively (Additional file 4: Table S2). Taken together, 15 CNVs were significantly associated with all three yield traits. One top-ranked CNV, CNV10 (chr5:9,756,491-9,837,147), reached significance values of 9.66 × 10^-12^, 8.38 × 10^-12^ and 2.23 × 10^-7^ for MY, FY, and PY, respectively. In the proximity of this region, two QTL effect peaks were reported at the 1 to 3 cM region of chr5 for milk traits in Holstein cattle using SNP data [11]. On chr14, we identified CNV1 (chr14:11,250,157-11,307,423) starting from SNP Hapmap29947-BTC-070181, near the previously reported milk production QTL regions. This CNV had the highest frequency (96.03%) and is 57,267 bp long. Approximately 6 Mb upstream of this CNV segment, the *NIBP* gene has been reported to also have highly significant effect for milk yield [8, 48]. Several important genes located further upstream, including *DGAT1* and *VPS28*, have been validated to have highly significant effects for milk production traits [12, 49]. Within the gene cluster including *DGAT1* and *NIBP*, our previous SNP-based study using U.S. Holstein cows also identified some SNPs, which are significantly associated with effects on milk production trait [8].

We also identified 11 and 29 CNVs that were significantly associated with FP and PP, respectively. The most significant CNV associated with FP was CNV33 (chr17:73,055,503-75,058,715) with a p-value of 7.00 × 10^-23^). We also found other CNVs like CNV12, CNV18, CNV28 and CNV34. However, CNV28 overlaps with only one QTL previously reported on chr7 [50]. Using PP, we identified the largest number (23) of significantly associated CNVs. Eleven of them were also significantly associated with all three yield traits (Table 1 and Additional file 5: Figure S3). The top five CNVs were CNV3, CNV6, CNV1, CNV12 and CNV9 based on their p values of the association test. QTL evidence from previous studies were also found in CNV1 [50–52], CNV24 [53], CNV26 [53, 54] CNV36 [55] and CNV37 [56–58].

### Relationship between associated CNVs and associated SNPs

**Figure 2:**
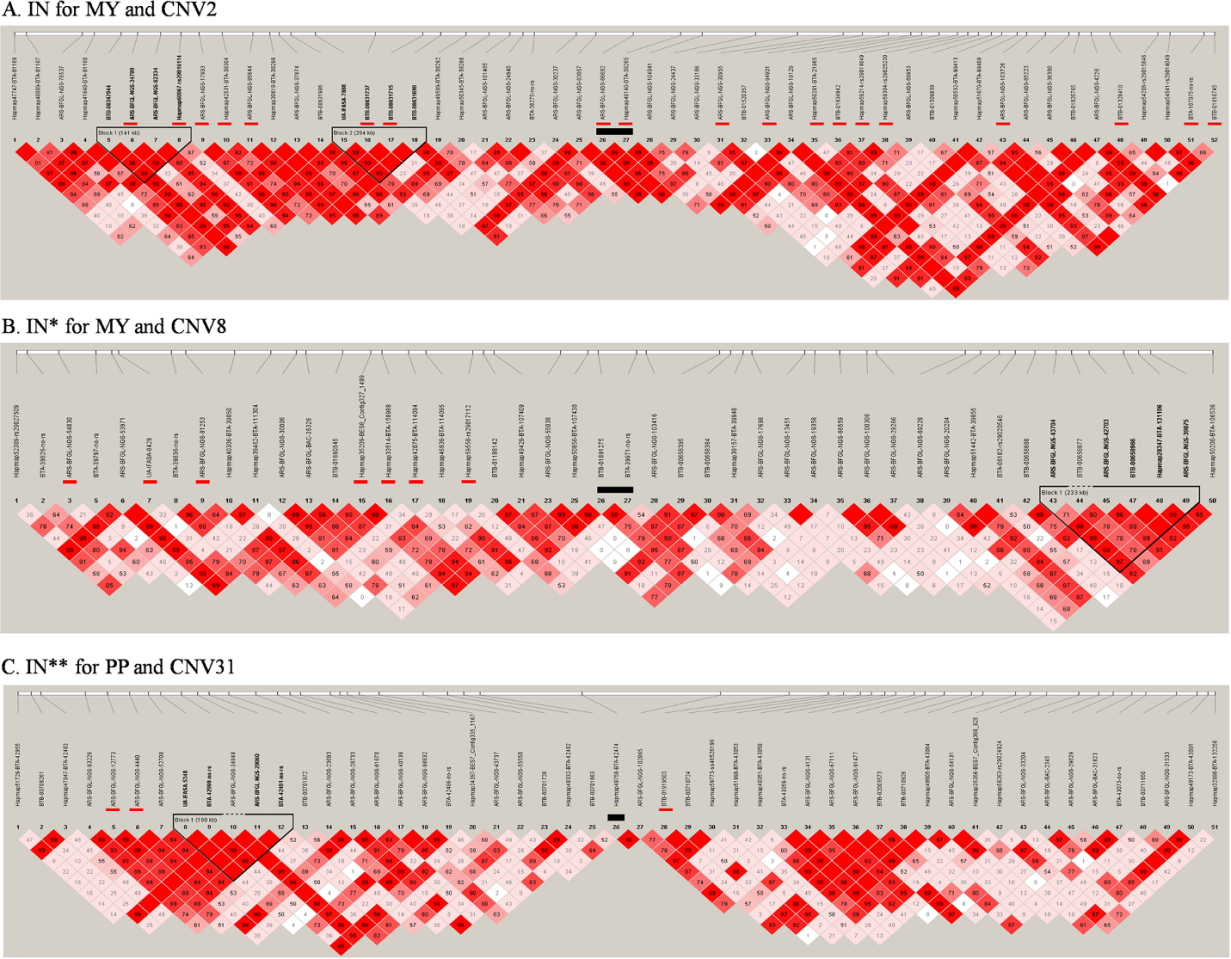
Haplotype analysis of (A) IN for MY, (B) IN* for MY and CNV8 and (C) IN** for PP and CNV31. Black bar represents CNV and red bars represent significant tag SNPs.

**Figure 3:**
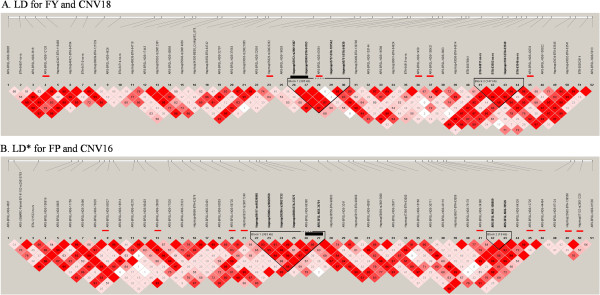
Haplotype analysis of (A) LD for FY and CNV18 and (B) LD* for FP and CNV16. Black bar represents CNV and red bars represent significant tag SNPs.

To further explore the relationship between SNPs and CNVs, we carried out association tests based on SNPs using the same 26,362 Holstein cattle SNP array data. Additional file 6: Table S3 contains SNP information near CNV regions. For all 82 possible combinations of traits and CNVs (less than 400 kb in length), we found 17 cases where significant SNPs directly overlapped with CNVs (e.g. IN for MY and CNV2, Figure 2A), 26 cases where significant SNPs were directly adjacent to CNVs (IN* for MY and CNV8, Figure 2B), and 14 cases where significant SNPs are the second SNPs next to CNVs (IN** for PP and CNV31, Figure 2C). To determine if there is any linkage between CNVs and neighboring SNPs, we performed LD analysis by computing pairwise D’ around CNV regions which included 25 SNPs both downstream and upstream of associated CNVs. Our results showed three cases (LD for FY and CNV18, Figure 3A) where CNVs were enclosed in the same haplotype block with at least one significant SNP, two cases where CNVs were directly adjacent to a haploblock which contains at least one significant SNP (LD* for FP and CNV16, Figure 3B), which suggested these CNVs could be represented by tagged SNPs within the same haploblock.

Some cases were complex situations involving both IN and LD. We generally assigned them to the IN, IN* and IN** instead of LD or LD* classes. However, no matter which class was used, CNVs in both the IN and LD classes were apparently well-captured by neighboring tag SNPs. It is interesting to note that although 9 out of 31 CNVs were located within breakpoints of haploblocks (Table 1 and Additional file 7: Figure S4), 7 of them were still tagged by neighboring SNPs. For example, we found one haploblock from Hapmap55972-rs29011387 to Hapmap50751-BTA-64830 in CNV28 with a length of 385 kb. We observed one SNP ARS-BFGL-NGS-63581 which was significantly associated with MY, FY, and PY (Additional file 7: Figure S4). We obtained one haploblock from Hapmap54599-rs29019617 to Hapmap48210-BTA-120730 in CNV21 and one SNPs Hapmap48210-BTA-120730 were detected to associate with MY, FY, PY and PP. For CNV32, one haploblock from ARS-BFGL-NGS-109612 to ARS-BFGL-NGS-26195 that contained a PY-associated SNP INRA-655 was identified. For CNV26, one large region containing three adjacent haploblocks (161 kb, 286 kb, 85 kb), which ranged from ARS-BFGL-BAC-28908 to ARS-BFGL-NGS-501, was found. These regions contained many SNPs associated with production traits. Additionally, two adjacent haploblocks were detected near CNV4, from ARS-BFGL-NGS-234 to ARS-BFGL-NGS-35131. This region contains one SNP ARS-BFGL-NGS-102090, which was significantly associated with MY, FY, and PY. Moreover, three adjacent haploblocks were found near CNV 28 (from ARS-BFGL-NGS-100845 to ARS-BFGL-NGS-13798), several significant associated SNPs were also found in this region.

**Figure 4:**
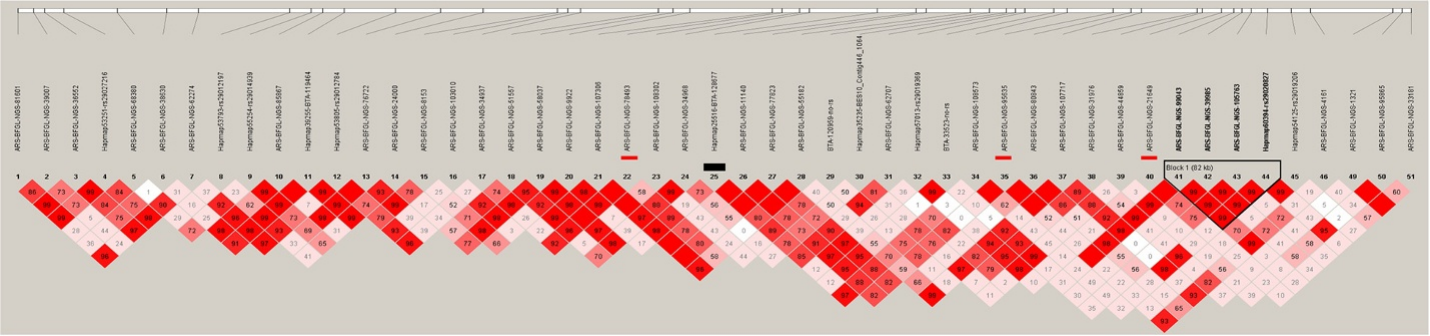
**Haplotype analysis of NN for PP and CNV6.** Black bar represents CNV and red bars represent significant tag SNPs.

Finally, we also found 20 cases where CNVs were not related (overlapping, neighboring or LD) with significantly associated SNPs (Table 3), suggesting that the impacts of those CNVs were probably not captured (e.g., NN for PP and CNV6, Figure 4. For more examples, please see Additional file 7: Figure S4). The possibility for this observation is that CNVs are likely to work as independent variants besides SNP. For example, we indentified a haploblock embedded in CNV16, which spanned 363 kb on chr27. However, no significant associated SNPs were found in this haploblock region for PY.

**Table 3:** **Summary of relationship between CNVs and SNPs**

	MY		FY		PY		FP		PP		All	
	Count	%	Count	%	Count	%	Count	%	Count	%	Count	%
NN	2	11.76%	2	10.53%	2	12.50%	2	28.57%	8	34.78%	16	19.51%
LD NS	2	11.76%	1	5.26%	1	6.25%	0	0.00%	0	0.00%	4	4.88%
Not tagged	4	23.53%	3	15.79%	3	18.75%	2	28.57%	8	34.78%	20	24.39%
LD	0	0.00%	1	5.26%	2	12.50%	0	0.00%	0	0.00%	3	3.66%
LD*	0	0.00%	0	0.00%	0	0.00%	1	14.29%	1	4.35%	2	2.44%
IN	4	23.53%	4	21.05%	3	18.75%	0	0.00%	6	26.09%	17	20.73%
IN*	6	35.29%	5	26.32%	5	31.25%	4	57.14%	6	26.09%	26	31.71%
IN**	3	17.65%	6	31.58%	3	18.75%	0	0.00%	2	8.70%	14	17.07%
Tagged	13	76.47%	16	84.21%	13	81.25%	5	71.43%	15	65.22%	62	75.61%
Total	17		19		16		7		23		82	

For the meanings of IN*, IN**, LD*, please refer to the main text.

## Conclusions

Previous and current genome wide association studies have been investigated to identify significant genes or linked markers based on SNPs. In this CNV-based study, our results indicate that CNV are associated with, and likely contribute to, differences in milk production. Our study provides a systematic estimate that approximately one-quarter of CNVs are not captured by LD with nearby SNPs. This provides an alternative framework to explain the missing heritability of complex traits. This study helps to fill gaps left by current SNP-based genome wide association and selection studies. Therefore, we conclude that combining CNV with SNP analyses reveals more genetic variations underlying milk production traits than can be revealed by SNPs alone. Interrogation of the genome for both CNVs and SNPs, including common and rare variations, could be an effective way to identify the causes of complex diseases and traits [16, 59]. A more comprehensive appreciation of the full spectrum of genetic variation may unravel the genetic basis for milk production.

### Availability of supporting data

Supporting information is available in the additional files and further supporting data is available from the authors on request.

## Electronic supplementary material

Additional file 1: Figure S1: Boxplot of reliability of five production traits. (PDF 76 KB)

Additional file 2: Figure S2: Heritability of five production traits. (PDF 75 KB)

Additional file 3: Table S1: List of CNV segments. (XLSX 25 KB)

Additional file 4: Table S2: Cattle QTL and RefSeq genes of 34 CNVs based on the UMD 3.1 assembly. (XLSX 20 KB)

Additional file 5: Figure S3: Overlapping relationships of 34 significantly associated CNV segments among five milk production traits. (PDF 107 KB)

Additional file 6: Table S3: SNPs near CNV regions. (XLSX 210 KB)

Additional file 7: Figure S4: Haplotype block views. (PDF 1 MB)
