# Supplementary material for: Genome wide CNV analysis reveals additional variants associated with milk production traits in Holsteins
Source: BMC Genomics. 2014 Aug 15;15(1):683. doi: 10.1186/1471-2164-15-683 (PMC4152564; doi:10.1186/1471-2164-15-683)
Supplement: Supplementary file 1 — Additional file 1: Figure S1: Boxplot of reliability of five production traits. (PDF 76 KB) [file 12864_2014_6385_MOESM1_ESM.pdf]

Additional file 4: Figure S1. Boxplot of reliability of five production traits.

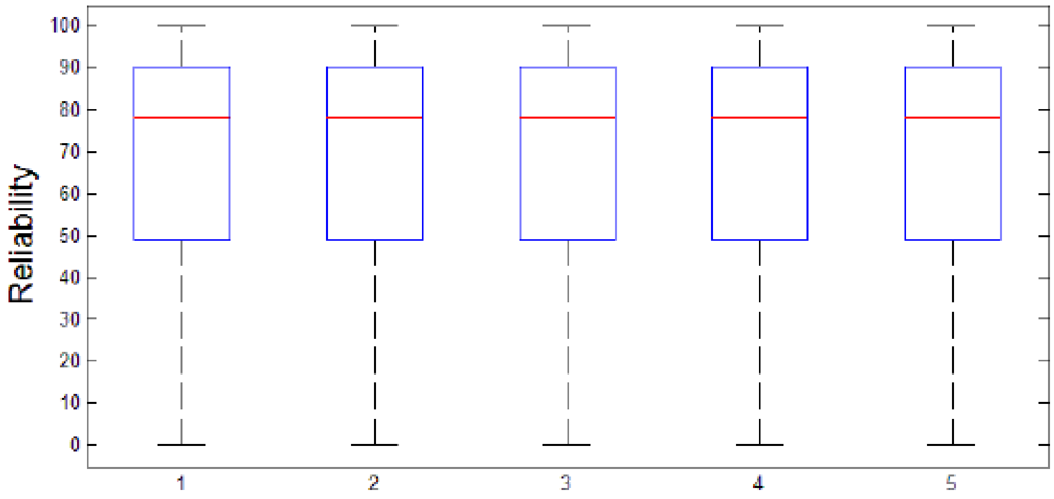

Figure S1. Boxplot of reliability of five production traits. From left to right were Milk Yield, Fat Yield, Protein Yield, Fat Percentage and Protein Percentage.
