# Supplementary material for: Genome wide CNV analysis reveals additional variants associated with milk production traits in Holsteins
Source: BMC Genomics. 2014 Aug 15;15(1):683. doi: 10.1186/1471-2164-15-683 (PMC4152564; doi:10.1186/1471-2164-15-683)
Supplement: Supplementary file 2 — Additional file 2: Figure S2: Heritability of five production traits. (PDF 75 KB) [file 12864_2014_6385_MOESM2_ESM.pdf]

Additional file 5: Figure S2. Heritability of five production traits.

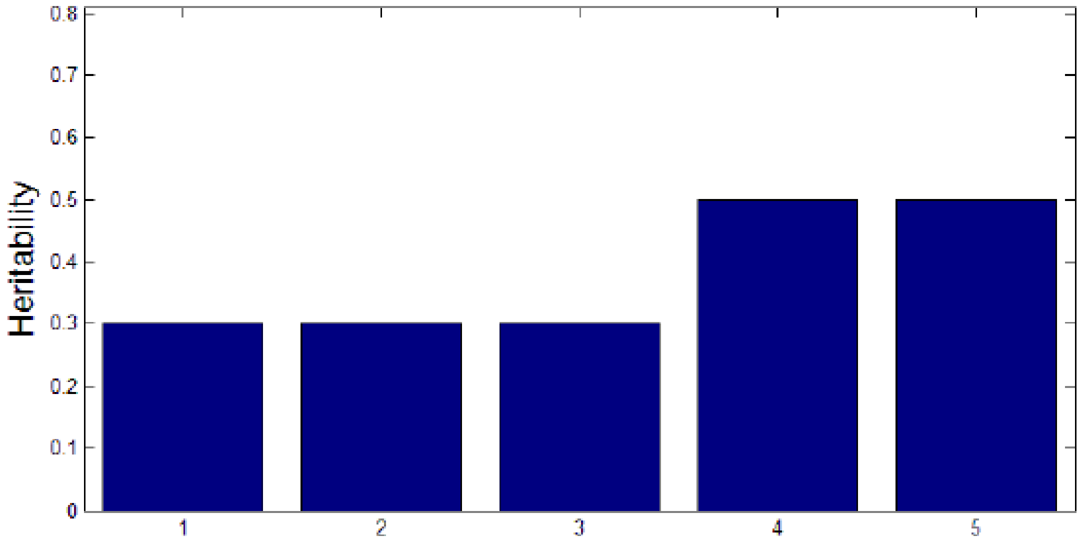

Figure S2. Heritability of five production traits. From left to right were Milk Yield, Fat Yield, Protein Yield, Fat Percentage and Protein Percentage.
