# Supplementary material for: Genome wide CNV analysis reveals additional variants associated with milk production traits in Holsteins
Source: BMC Genomics. 2014 Aug 15;15(1):683. doi: 10.1186/1471-2164-15-683 (PMC4152564; doi:10.1186/1471-2164-15-683)
Supplement: Supplementary file 5 — Additional file 5: Figure S3: Overlapping relationships of 34 significantly associated CNV segments among five milk production traits. (PDF 107 KB) [file 12864_2014_6385_MOESM5_ESM.pdf]

Additional file 6: Figure S3. Overlapping relationships of 34 significantly associated CNV segments among five milk production traits.

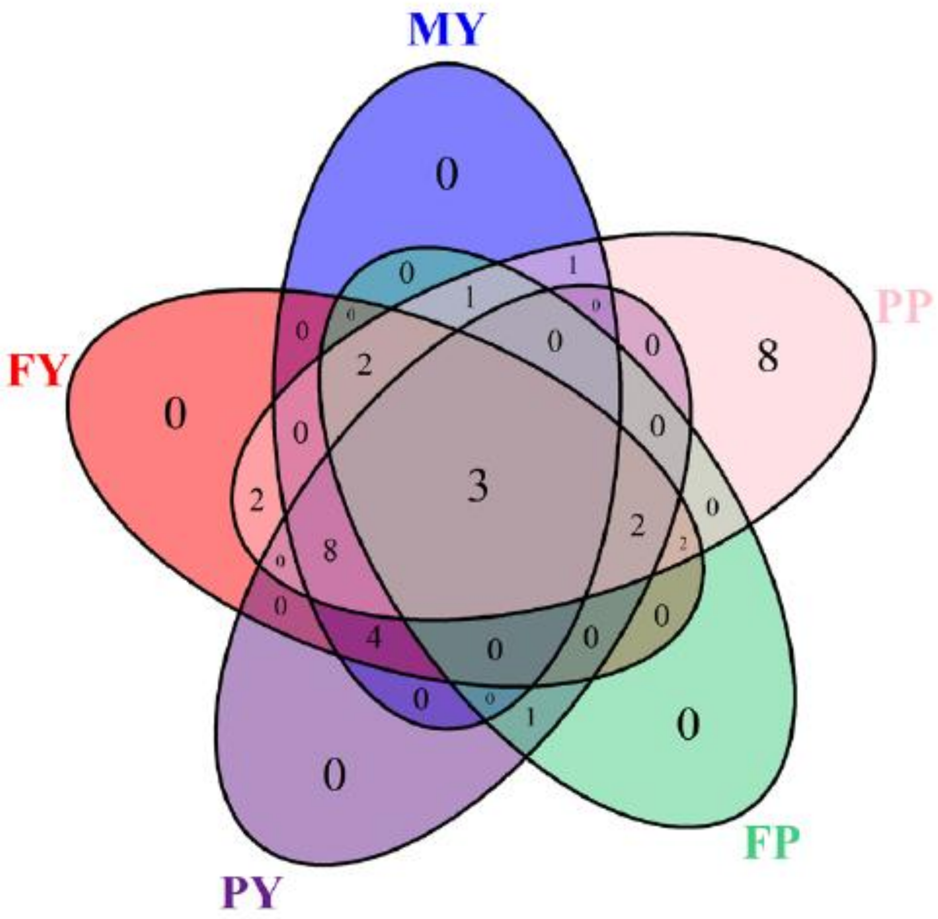

Figure S3. The overlapping relationships of 34 significantly associated CNV segments among five milk production traits: Milk Yield (MY), Fat Yield (FY), Protein Yield (PY), Fat Percentage (FP) and Protein Percentage (PP).
