# Supplementary material for: Genome wide CNV analysis reveals additional variants associated with milk production traits in Holsteins
Source: BMC Genomics. 2014 Aug 15;15(1):683. doi: 10.1186/1471-2164-15-683 (PMC4152564; doi:10.1186/1471-2164-15-683)
Supplement: Supplementary file 7 — Additional file 7: Figure S4: Haplotype block views. (PDF 1 MB) [file 12864_2014_6385_MOESM7_ESM.pdf]

Additional file 7: Figure S4. Haplotype block views.

CNV1

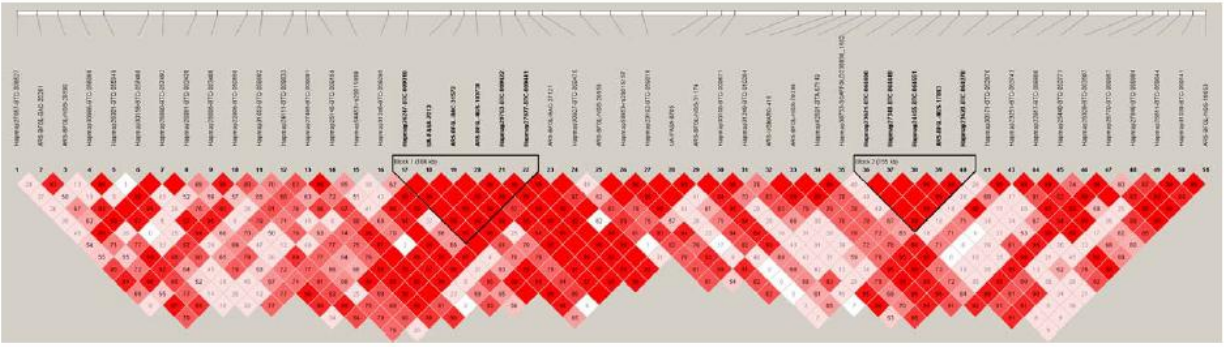

CNV2

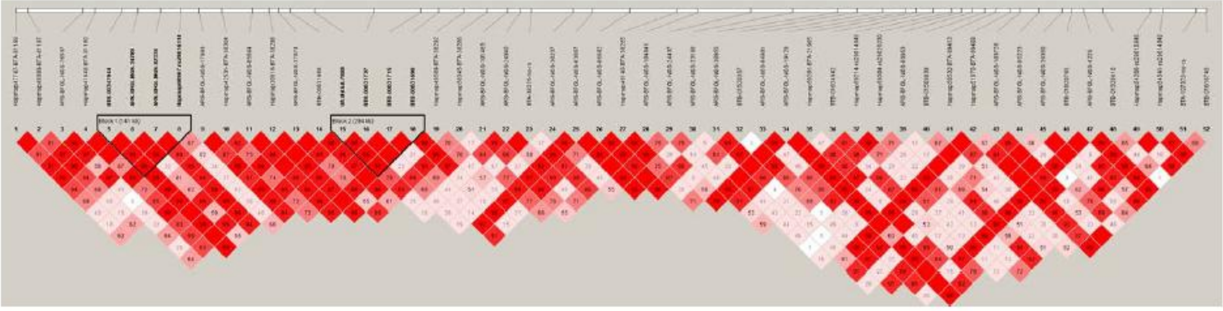

CNV3

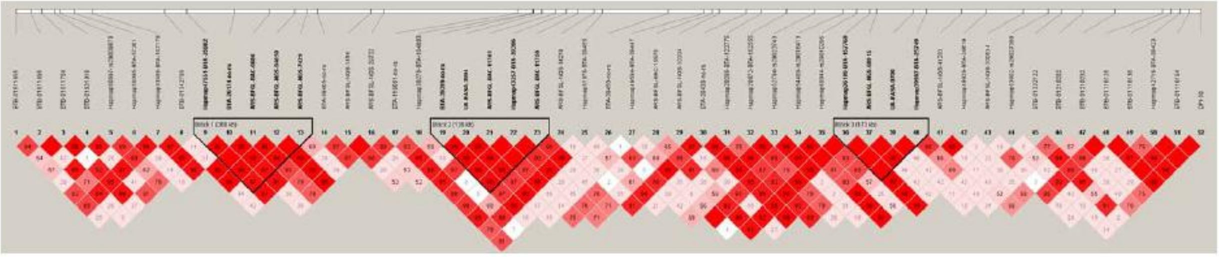

CNV4

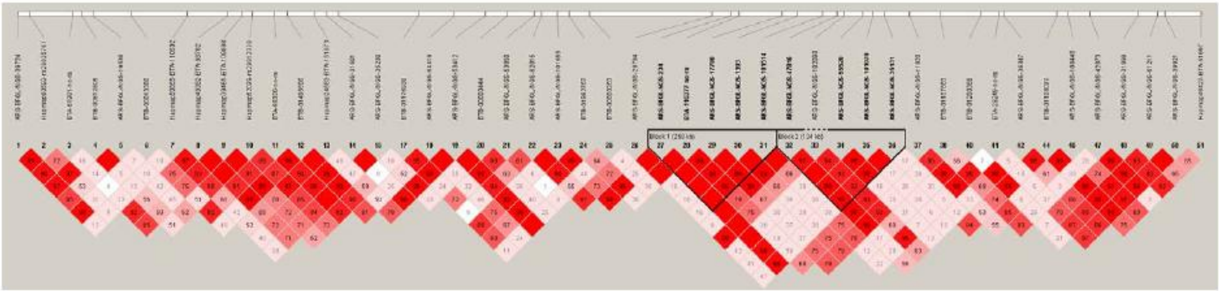

CNV5

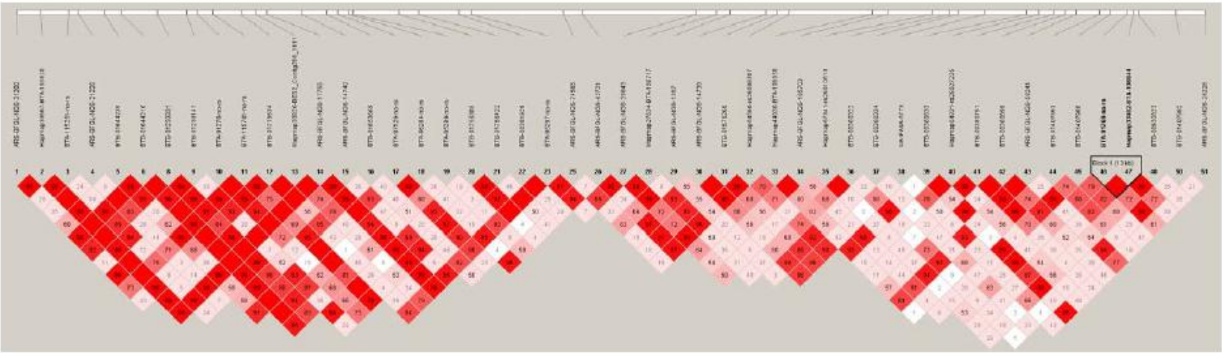

CNV6

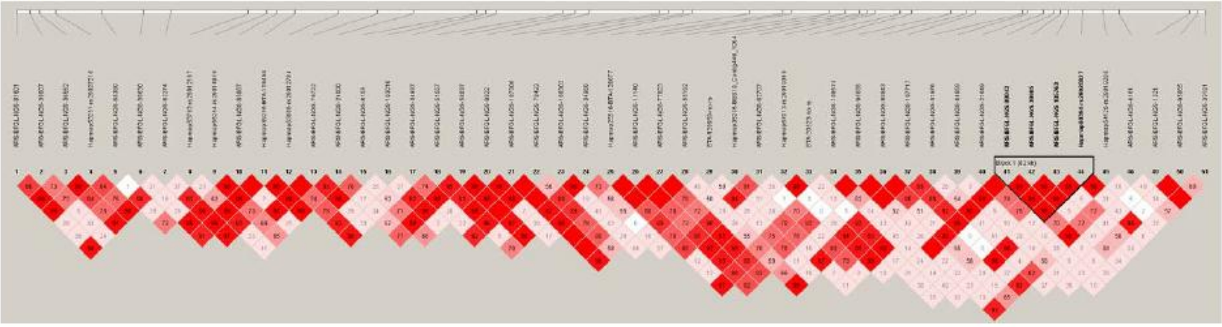

CNV7

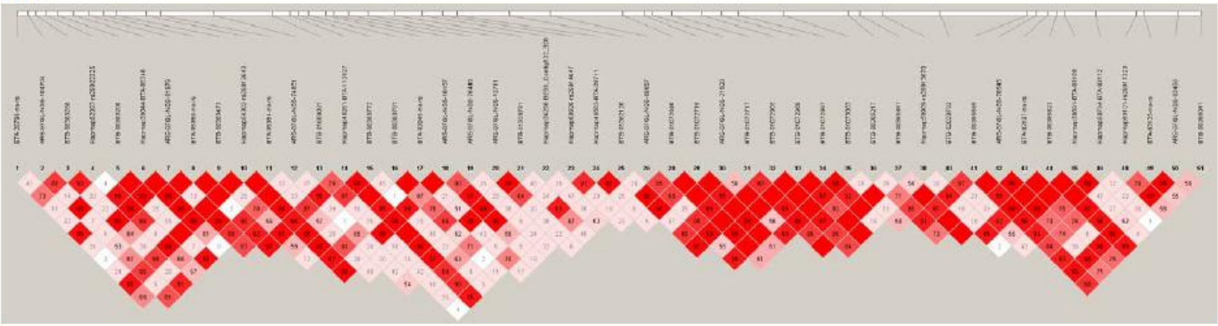

CNV8

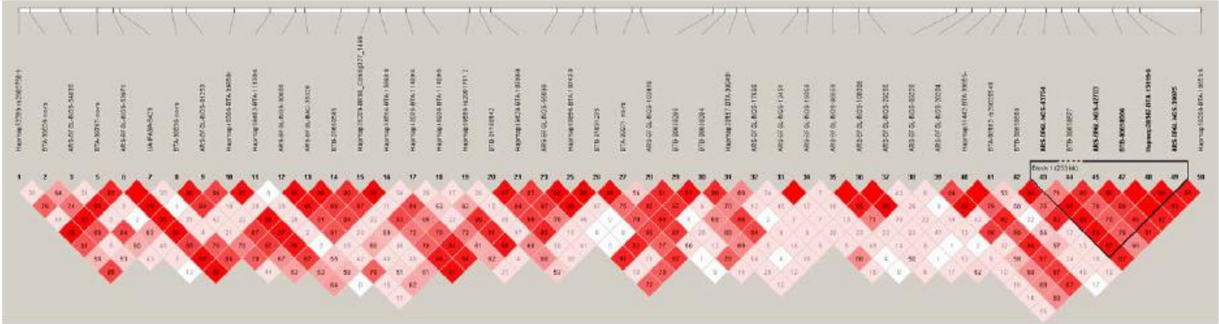

CNV9

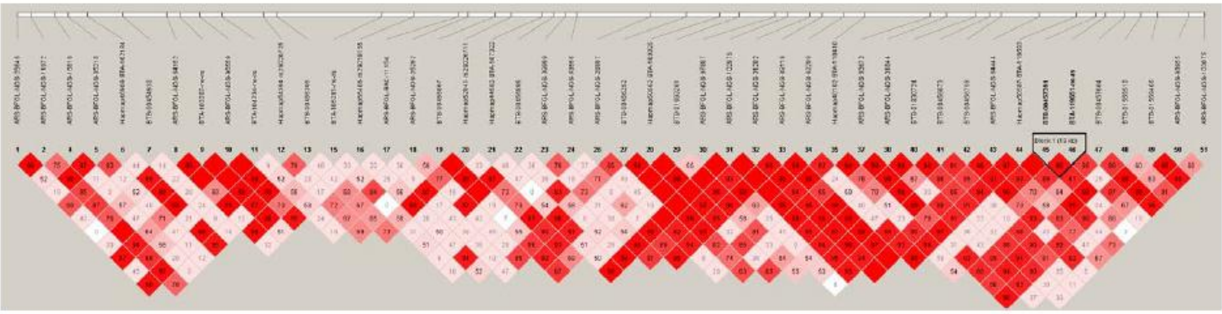

CNV10

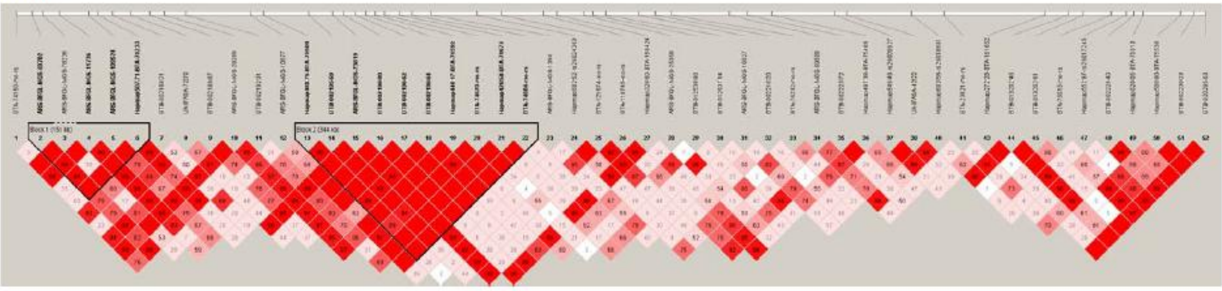

CNV11

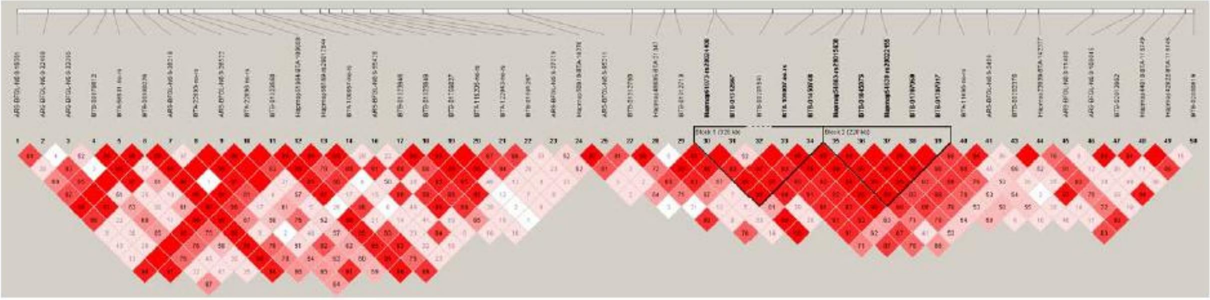

CNV12

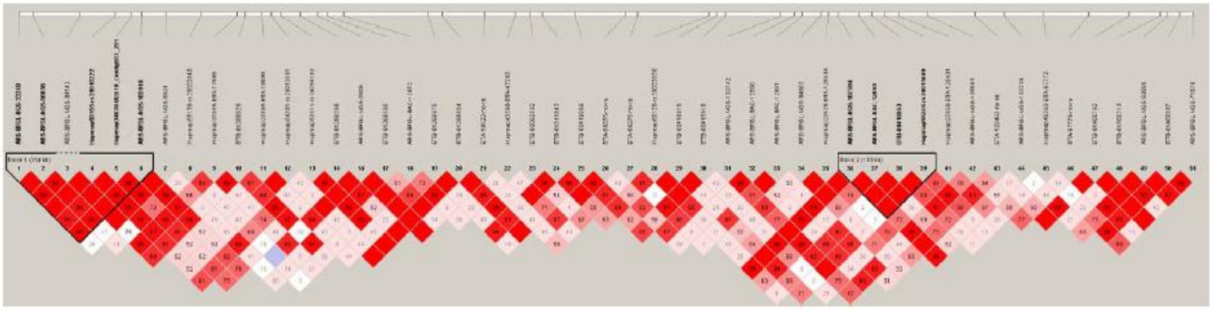

CNV13

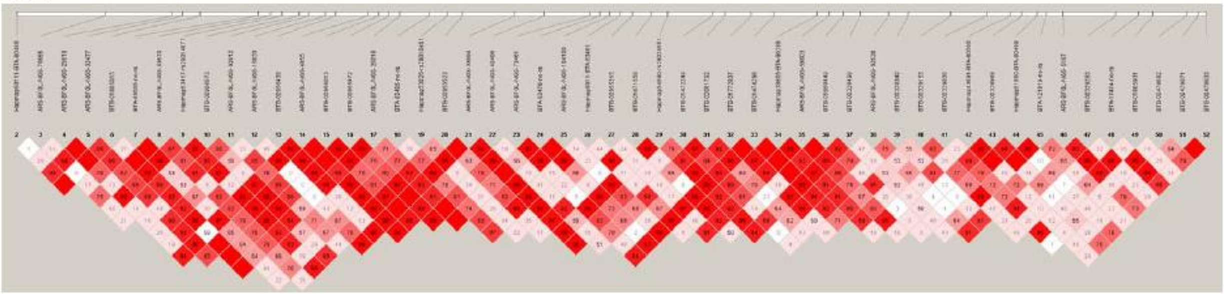

CNV14

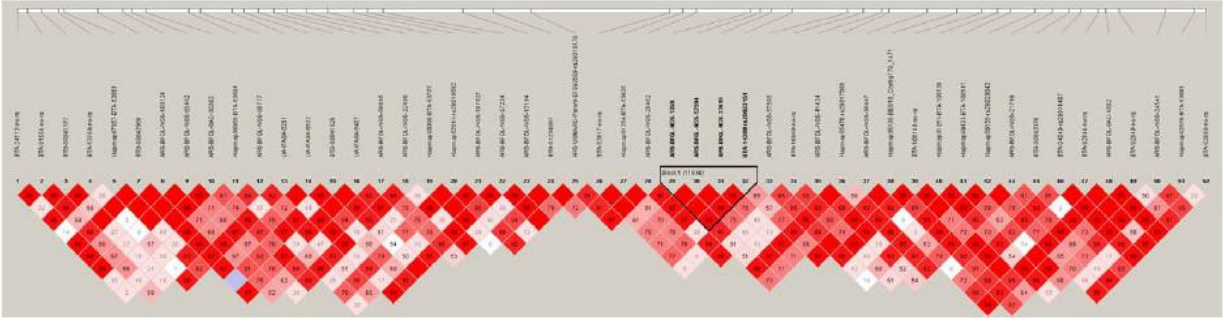

CNV15

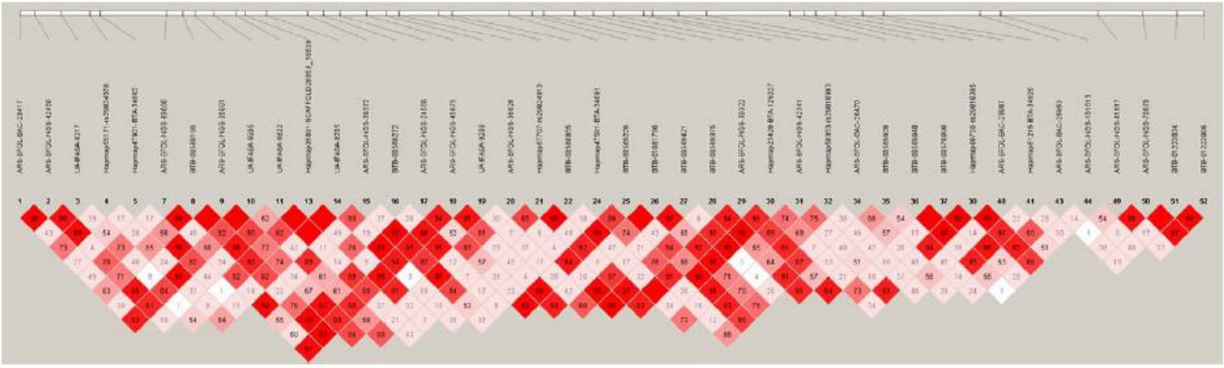

CNV16

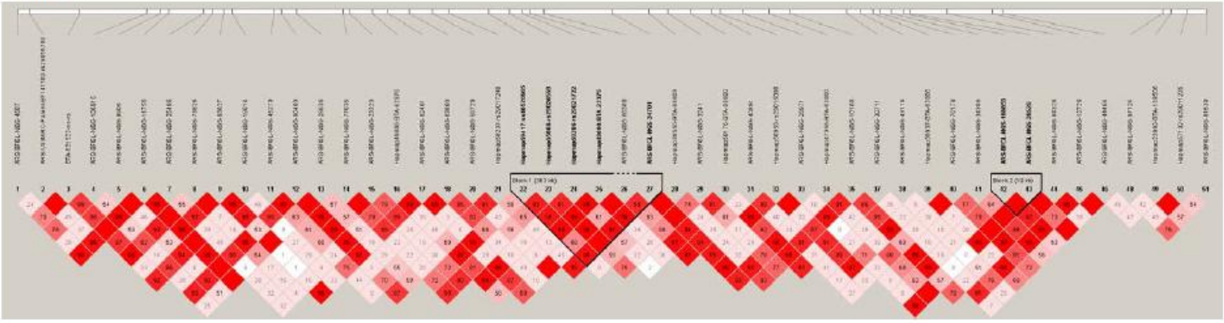

CNV17

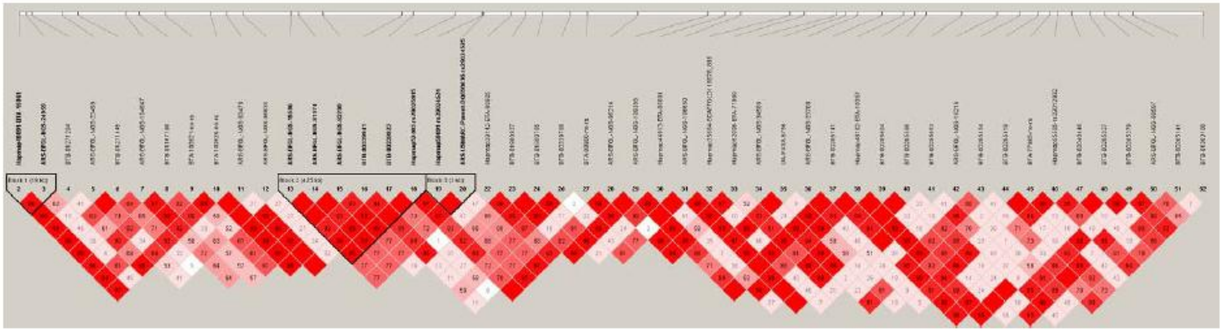

CNV18

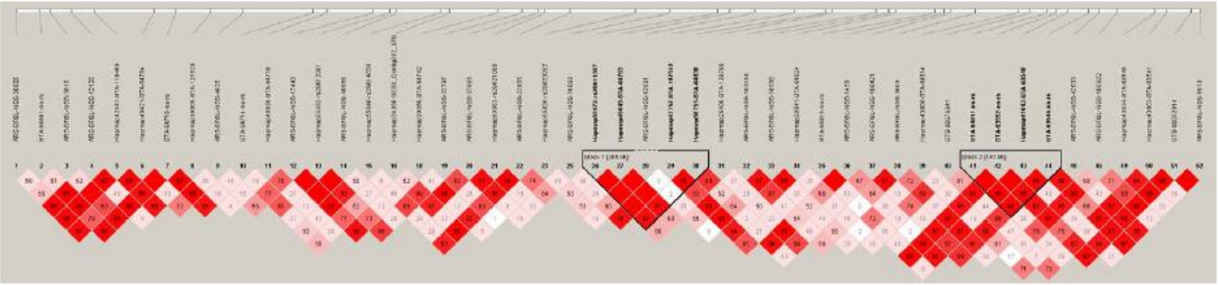

CNV19

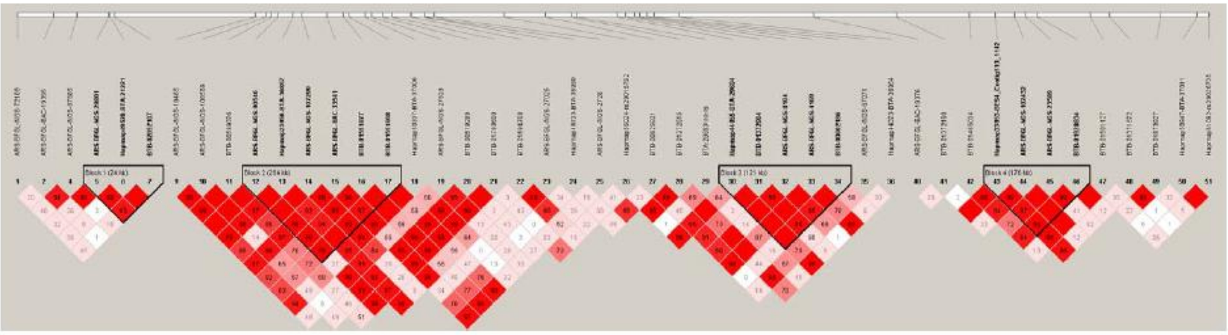

CNV20

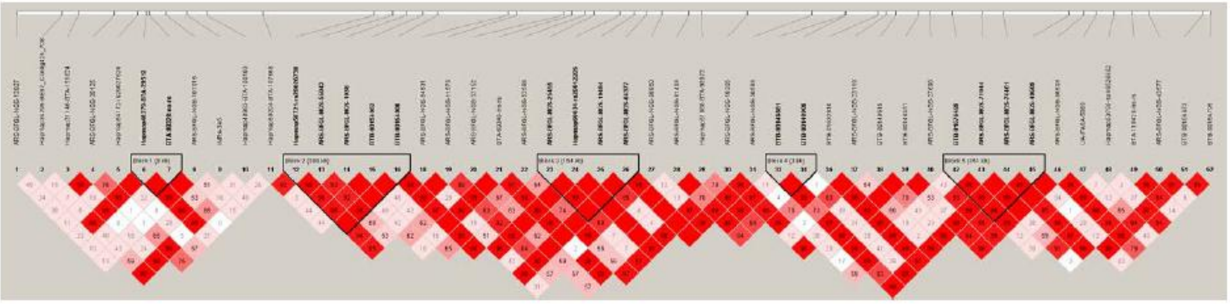

CNV21

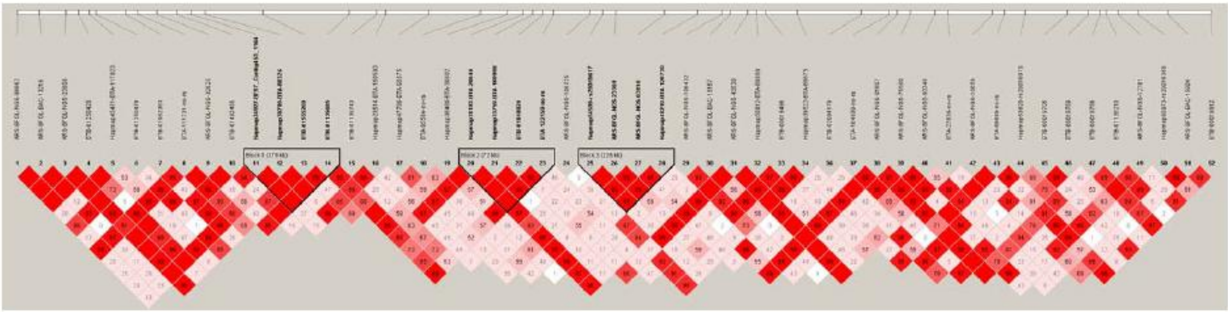

CNV22

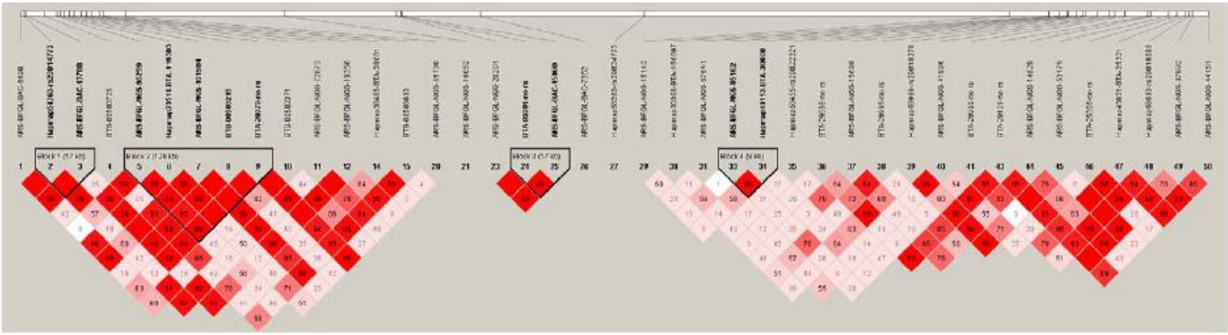

CNV23

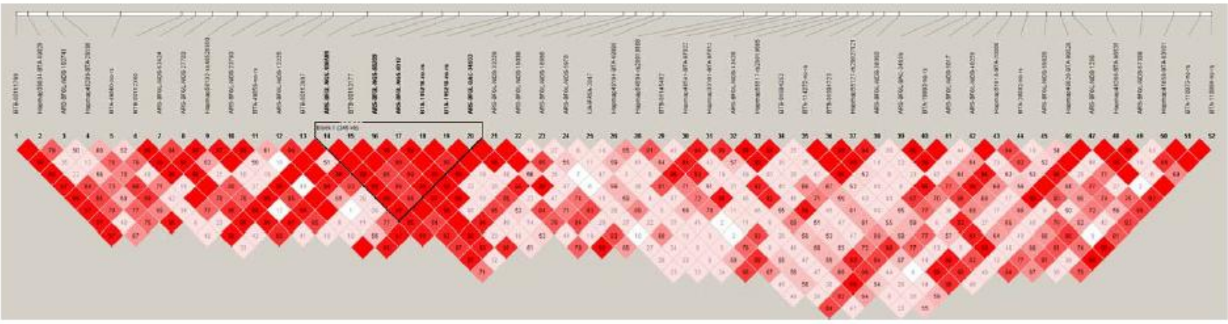

CNV24

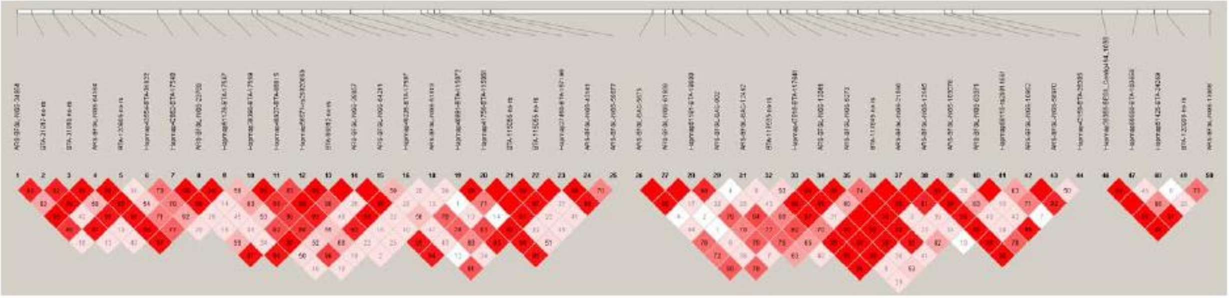

CNV25

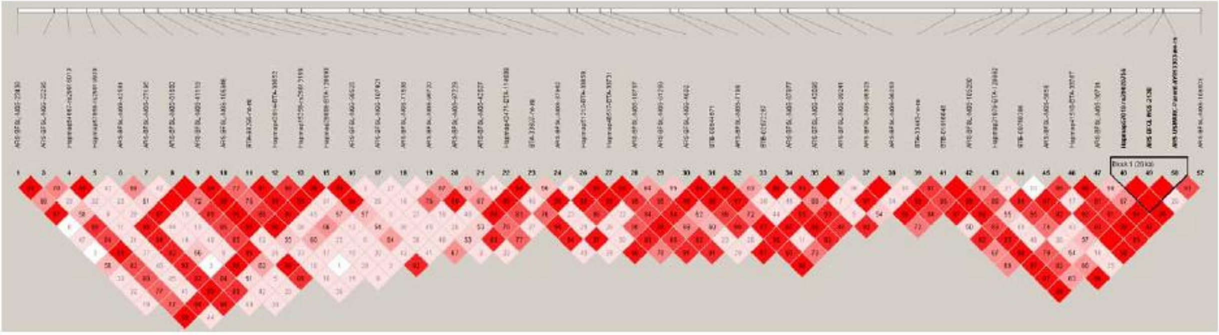

CNV26

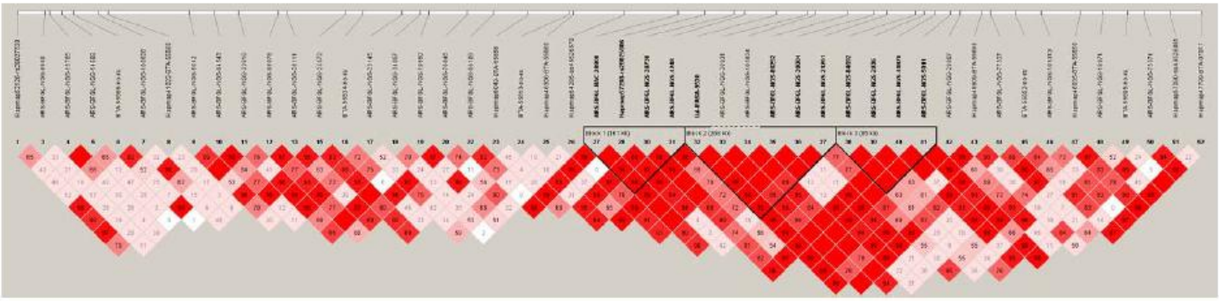

CNV27 was skipped due to its large size.

CNV28

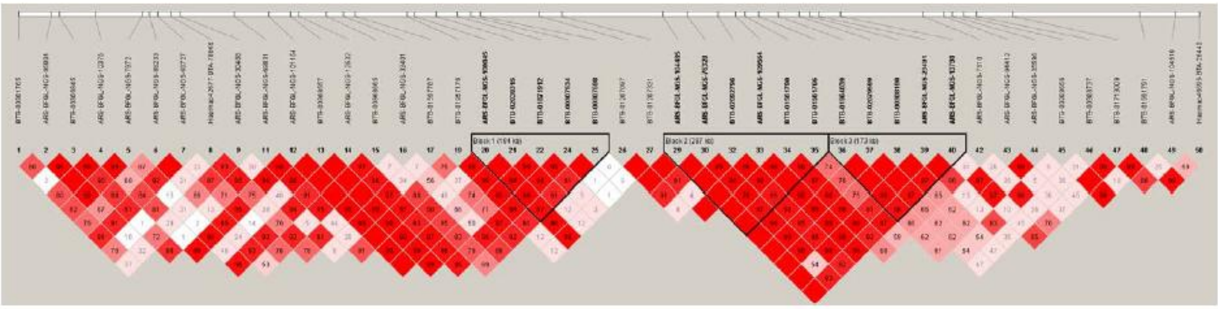

CNV29

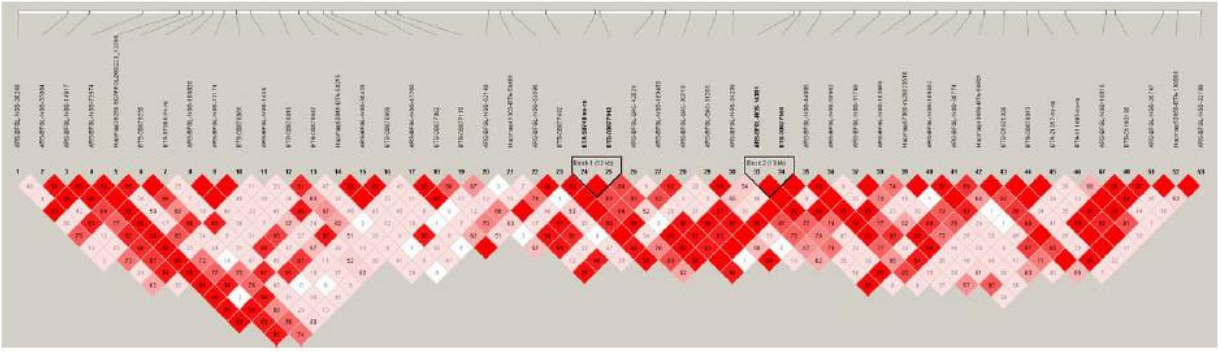

CNV30

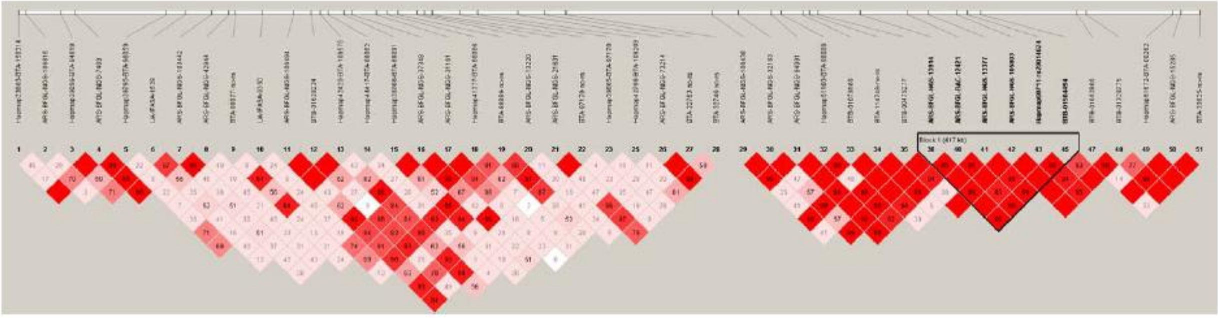

CNV31

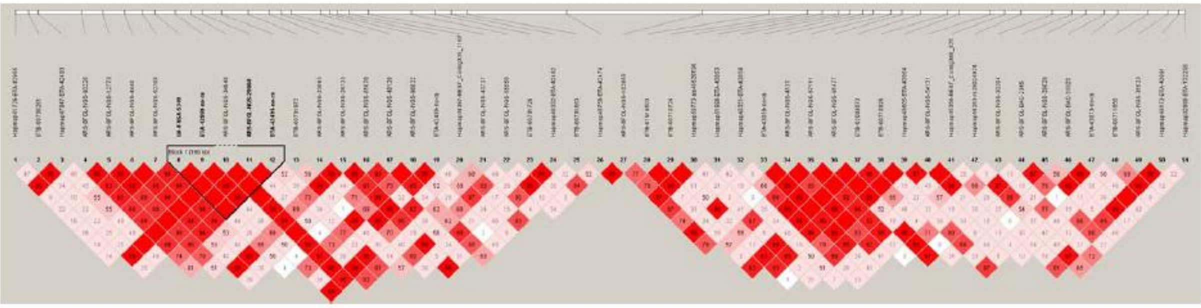

CNV32

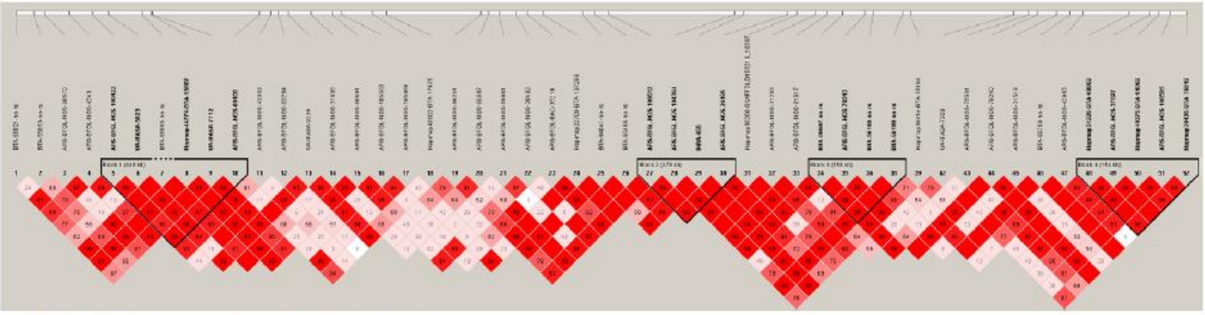

CNV33 to CNV37 were skipped due to their large sizes.
